# Insertion of metal cations into hybrid organometallic halide perovskite nanocrystals for enhanced stability: eco-friendly synthesis, lattice strain engineering, and defect chemistry studies†

**DOI:** 10.1039/d2na00053a

**Published:** 2022-05-12

**Authors:** Mohammed Nazim, Aftab Aslam Parwaz Khan, Firoz Khan, Sung Ki Cho, Rafiq Ahmad

**Affiliations:** Department of Chemical Engineering, Kumoh National Institute of Technology 61 Daehak-ro, Gumi-si Gyeongbuk-do 39177 Republic of Korea nazimopv@gmail.com; Department of Energy Engineering Convergence, Kumoh National Institute of Technology 61 Daehak-ro, Gumi-si Gyeongsangbuk-do 39177 Republic of Korea; Chemistry Department, Faculty of Science, King Abdulaziz University P. O. Box 80203 Jeddah 21589 Saudi Arabia aapkhan@gmail.com; Interdisciplinary Research Center for Renewable Energy and Power System (IRC-REPS), King Fahd University of Petroleum & Minerals (KFUPM) Dhahran 31261 Saudi Arabia; Centre for Nanoscience and Nanotechnology, Jamia Millia Islamia New Delhi-110025 India rahmad5@jmi.ac.in

## Abstract

In this work, we developed a facile and environmentally friendly synthesis strategy for large-scale preparation of Cr-doped hybrid organometallic halide perovskite nanocrystals. In the experiment, methylammonium lead bromide, CH_3_NH_3_PbBr_3_, was efficiently doped with Cr^3+^ cations by eco-friendly method at low temperatures to grow crystals *via* antisolvent-crystallization. The as-synthesized Cr^3+^ cation–doped perovskite nanocrystals displayed ∼45.45% decrease in the (100) phase intensity with an enhanced Bragg angle (2*θ*) of ∼15.01° compared to ∼14.92° of pristine perovskites while retaining their cubic (221/*Pm-cm*, ICSD no. 00-069-1350) crystalline phase of pristine perovskites. During synthesis, an eco-friendly solvent, ethanol, was utilized as an antisolvent to grow nanometer-sized rod-like crystals. However, Cr^3+^ cation-doped perovskite nanocrystals display a reduced crystallinity of ∼67% compared to pristine counterpart with ∼75% crystallinity with an improved contact angle of ∼72° against water in thin films. Besides, as-grown perovskite nanocrystals produced crystallite size of ∼48 nm and a full-width-at-half-maximum (FWHM) of ∼0.19° with an enhanced lattice-strain of ∼4.52 × 10^−4^ with a dislocation-density of ∼4.24 × 10^14^ lines per m^2^ compared to pristine perovskite nanocrystals, as extracted from the Williamson–Hall plots. The as-obtained stable perovskite materials might be promising light-harvesting candidates for optoelectronic applications in the future.

## Abstract 2

We developed a facile and eco-friendly synthesis strategy for large-scale preparation of undoped and Cr-doped hybrid organometallic halide perovskite nanocrystals.

## Graphical abstract

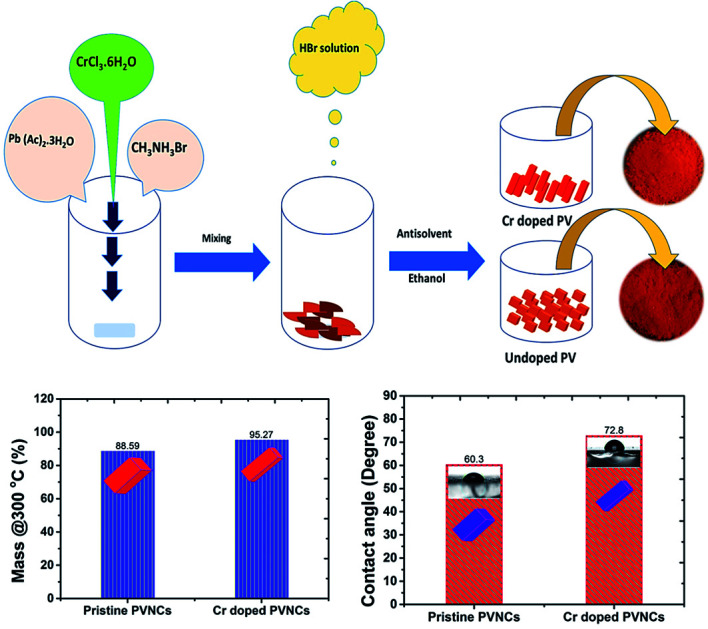

## Introduction

The development of a facile, large-scale and cost-effective technique is the need of the hour to provide clean energy and reduce global warming or climate change problems with growing energy demands.^1,2^ Renewable energy techniques have been applied to use multi-dimensional hybrid organic–inorganic perovskite nanocrystals in materials science, chemistry, physics, and engineering.^3,4^ Additionally, semiconducting hybrid perovskite nanocrystals have been widely attracting attentions due to their ease of synthesis, excellent environmental stability and unique structures compared to perovskite bulk materials, which possess high doping probability or replacement of organic and inorganic components to tune their bandgap energy at various chemical compositions.^5,6^ In the last decade, lead halide hybrid perovskite materials have emerged as the strongest materials to achieve the best performance of ∼25.2% for photovoltaic devices with improved chemical, thermal and photostability in air atmosphere.^7–9^ In general, semiconductor perovskite materials exhibit reorientation of organic-cations, low exciton binding energies, and high charge mobilities to support the charge-transport mechanism for vast applications such as light detection, light emission, and photoelectrochemical solar water-splitting for thin-film devices.^9–11^

To fulfil the growing energy demand, a bulk architecture of semiconducting quantum dots (SQDs) with organic materials demonstrates various structural geometries such as zero-, one- and two-dimensional nanomaterials for light-emitting diodes (LEDs), photodetectors (PDs), photovoltaics (PVs), and sensor applications.^12–14^ In addition, semiconducting nanomaterials with <20 nm size have been uniquely featured to establish a structure–property relationship, which tunes optical and photoelectric properties due to quantum-confinement effects in light–matter interactions for energy applications.^11,15^ The halide perovskite nanocrystals (PVNCs) display extraordinary properties including broad absorption capability in the solar spectrum, a huge optical absorption coefficient, and well-matched electronic energy states with low-cost production in a short time period.^14,15^ In semiconducting materials, halide perovskite nanocrystals have gained huge interests by modifying their tolerance factor (*t*), resulting in structural-defects, which tune their optical and electronic features compared to conventional PVNCs.^16,17^ Recently, PVNCs have emerged as the most efficient electronic materials with excellent physico-chemical and photovoltaic properties. Due to diverse probability in shape and size, the nanomaterials might control their crystal phases during crystal growth based on reaction conditions such as solvents, reaction time, and temperature.^18,19^ Compared to metal oxides, PVNCs have been reported in limited studies to progress *via* controlled growth based on their phases, facets, sizes, and shapes, but no optimized technique has been available to date.^20,21^ In spite of enormous approaches, an optimized synthesis procedure is still a big challenge for researchers in the precise control of PVNCs with phase calibration and crystal geometry.^22–24^ Basically, the crystal-growth reactions of PVNCs showed a short time to make its reaction-mechanism really tough to understand with a controlled facet, design and shape till now.

On a great concern to stability, the chemical reactivity of PVNCs with air and moisture is a major hurdle for their long-term and commercial applications due to their decomposition features upon heat and light irradiation during harsh environmental conditions.^25,26^ Furthermore, the defect and strain of perovskite materials have been significantly relieved by various encapsulation techniques to suppress ion-migration or high density of defects due to solution-processed fabrication at low temperatures.^27–29^ In addition, semiconducting perovskite materials have been presenting many tunable properties using defect-engineering *via* chemical-doping, which introduces novel functional sites to control their physical and optoelectronic properties.^28–30^ During crystal growth, perovskite materials have a tunable lattice-strain due to chemical doping, solvent washing, thermal treatment and anti-solvent treatment for crystallization, which lead to improve fluorescence emission properties, charge mobility and electrical conductance.^31,32^ Furthermore, the ionic migration and accumulation of reactive species might create more defects in the crystal structure as well as adjacent functional layers, resulting in performance degradation of optoelectronic devices.^33,34^ Interestingly, the tunable energy band-gap of PVNCs makes them light-harvesting materials for efficient optical devices due to broad absorption in visible region based on different compositions of PVNCs.^35,36^ In continuation, the partial replacement of organic or inorganic components might affect the crystal distortion to reduce growth rate of crystals during crystallization.^37^ Herein, the antisolvent crystallization technique, a facile, and cost-effective approach, was adopted to control nucleation of crystals resulting in diverse crystal shapes and morphological phases. The partial replacement of Pb^2+^ cations (ionic radius of 119 pm) was observed by small-sized Cr^3+^ cations (ionic radius of 62 pm) with a high spin (t^3^_2g_e^0^_g_) electronic configuration due to partially filled orbitals in an octahedral environment. Recently, highly fluorescent dual metal (Cr^3+^ and Mn^2+^) cation doped cesium-based perovskite nanomaterial, (Cr^3+^:CsPbCl_3_:Mn^2+^) as a quantum dot (QD) has been reported as light-harvesting material with ultraviolet absorption, longer Stokes shift, and excellent stability for solar cell applications.^38^ On Cr^3+^ cation (∼7.5 mol percent) doping, an excellent photoluminescence quantum yield (PL-QY) of ∼97% was achieved due to the synergistic effect of high energy transfer and passivation effect of Cr^3+^ cations, resulting in a superior performance of ∼22.35%.

Herein, Cr^3+^ cations as dopants were applied to grow metal-doped hybrid organic-inorganic metal halide perovskite nanocrystals (HOME PVNCs) with tuned optical, physical, structural, and crystal lattice properties. In the present paper, a cost-effective eco-friendly synthesis strategy has been used to grow HOME PVNCs as light-harvesting materials with or without metal ion doping which improve their thermal stability and tune strain properties for diverse applications under ambient conditions. It was revealed that smooth insertion of small-sized Cr^3+^ cations partially replaces Pb^2+^ cations of perovskite nanocrystals to boost their optical properties estimated in terms of Tauc plots and Urbach plots with high thermal-stability along with detainment of cubic geometry. Specifically, perovskite nanocrystals might suppress surface grain boundaries and recombination losses *via* crystal defects after metal cation doping. Hence, Cr^3+^ cation-doping might produce novel doped-organometallic halide perovskite rod-like nanocrystals with cubic-phase retention with slightly modified crystal lattice parameters. Owing to such excellent properties, the as-obtained doped nanocrystals might be largely applicable in various optoelectronic fields in the future.

## Experimental

### Materials

For all the experiments, the chemicals and reagents were obtained from commercial sources and used without further purification. Lead(ii) acetate (Pb(OCH_3_COO)_2_, 99.99%), hydrogen bromide (HBr, 99.99%), dimethylformamide (DMF, 99.9%), ethanol (75%), anhydrous toluene (99.8%), and *n*-hexane (99.5%) were purchased from Sigma-Aldrich Company, Republic of Korea.

### Characterizations

X-ray diffraction (XRD) analysis was performed using an X-ray diffractometer (Bragg's angle range of 5–80°, Empyrean, Panalytical, USA) having a wavelength of Cu Kα radiation (*λ* = 1.5406 Å) for structure and bonding phases of perovskite materials. For perovskite films, UV-visible spectral analysis was measured (200−800 nm wavelength) on pristine ITO glass substrates using an Agilent Technologies instrument (Carey 5000 UV-vis-NIR spectrophotometer) equipped with a light source of deuterium and Tungsten lamps. In addition, field-effect scanning electron microscopic (FESEM) analysis was performed with perovskite crystals using a Hitachi instrument (SU 8230) in terms of high- and low-resolution images. Energy-dispersive X-ray spectroscopic (EDX) analysis was performed to find out doping weight-percentage (wt%) of metal components and their homogeneity in nanocrystals. While a Raman spectrometer (at *λ*_ex_ 532 nm) was used to investigate various bands using a Nicolet Almeca XRA instrument of Thermo Fisher Scientific Company Ltd. The thermal properties of perovskite nanocrystals were investigated using a thermogravimetric analysis instrument (TA Instruments-Waters, Korea, Auto Q500) in 25–600 °C temperature range. A differential scanning calorimetry (TA instruments, Discovery DSC) instrument was applied to find various phase changes of perovskite materials before and after metal doping. X-ray photoelectron spectroscopic (Thermo Fisher Scientific, Escalab 250Xi, USA) instrument display various binding interactions with an Al Kα X-ray wavelength of ∼1486.6 eV (monochromatic) under ultrahigh vacuum for pristine and doped PVNCs. The crystalline phases and intermolecular distance were evaluated using a transmission electron microscope (TEM, S-4800, Hitachi, Japan) in image and FFT patterns analysis for PVNCs.

### Eco-friendly approach of pristine MAPbBr_3_ nanocrystals

For cost-effective lead precursors, lead acetate trihydrate (Pb(CH_3_COO)_2_·3H_2_O) was utilized in place of its bromide counterpart. However, a strong complexing agent, trihydrate lead salt, presents a complicated chemistry responsible for perovskite conversion in presence of water.^39,40^ In summary, the stoichiometric PVNCs formed as follows:

1 Pb(AcO)2.3H_2_O + CH_3_NH_3_Br + HBr → CH_3_NH_3_PbBr_3_ + H_2_O

For synthesis, the hybrid perovskite precursor solution was prepared by mixing of Pb(OAc)_2_ and MABr in 1 : 1 ratio followed by stirring and addition of ∼400 μl of HBr solution to form an orange-yellow color slurry under ambient conditions. Then, pristine nanocrystals were grown *via* antisolvent crystallization by addition of ∼10 ml of ethanol in a stirred solution for 12 h. Thus, Cr^3+^-doped PVNCs were cleaned twice with ethanol and then dried at 80 °C temperature for up to 12 h to evaporate excess solvents.

### Eco-friendly synthesis of Cr doped MAPbBr_3_ nanocrystals

**Fig. 1:**
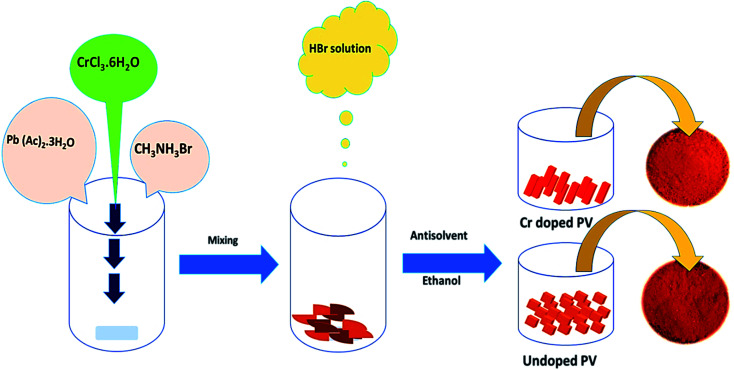
Schematic diagram of the eco-friendly synthesis of pristine and Cr-doped perovskite nanocrystals.

The hybrid perovskite precursor solution was prepared by mixing Pb (OAc)_2_ and MABr in 1 : 1 ratio, and appropriate amounts of Cr^3+^ cations (formulated as CH_3_NH_3_Cr_(*x*)_Pb_(1−*x*)_Br_3_) were mixed with stirring to form a brown slurry. Different amounts (0, 2.5, 5.0, and 7.5%) of hydrated chromium salt (Cr(iii)Cl_3_·6H_2_O) were added for experimental comparisons, and Pb^2+^ cations were partially replaced with Cr^3+^ cations. Subsequently, a HBr solution (∼37%, ∼400 μl) was dropwise added to above-mentioned solution with stirring at room temperature. Then, an antisolvent, ethanol (∼10 ml), was added and stirred for 1 h under ambient conditions. Thus obtained doped perovskite solution was heated at ∼80 °C for 12 h to develop Cr-doped PVNCs as orange crystals. The as-obtained Cr^3+^-doped PVNCs were further washed several times with ethanol and dried at ∼80 °C for 6 h to evaporate excess solvent to get ultrapure Cr-doped PVNCs (Fig. 1).

### Fabrication ofMAPbBr_3_ nanocrystals

The indium tin oxide (ITO) glass substrates were treated for sonication with detergent, acetone, water and isopropyl alcohol for up to ∼10 min each. The as-obtained perovskite materials were taken in appropriate amounts to make up precursor solutions in an anhydrous *N*,*N*-dimethylformamide (DMF) solvent for pristine and doped sample nanocrystals. The precursor perovskite solutions were applied for thin film deposition by a drop-casting method for doped MAPbBr_3_ in DMF solutions in ambient atmosphere. The as-obtained thin films were treated at ∼100 °C for 10 min to get orange perovskite thin films in ambient air.

## Results and discussion

### Thermal analysis of perovskite nanocrystals

In hybrid perovskite-based research, the anti-solvent crystallization technique was applied widely due to their promising, rapid and excellent synthetic style to achieve large-scale production for wide-area fabrication devices. Methylammonium (MA)-based perovskite (as MABX_3_, X = Br) nanocrystals display excellent thermal and air stability due to their high thermal capacity with no phase transitions up to ∼200 °C.^62^ The degradation of perovskite is based on hydration of perovskite crystals to form mono-hydrate in a reversible reaction under humid conditions.^41^ Thus, PbBr_2_ might precipitate as a solid to rule out any possibility of re-formation of perovskite nanocrystals. In addition, water is a strong polar solvent, and forms quite stable complexes with hybrid perovskite components.^42^ In TGA plots, organic and inorganic components have been decomposed and interpreted for perovskite precursors of hybrid CH_3_NH_3_PbBr_3_ nanocrystals as follows:

2 CH_3_NH_3_PbBr_3_(s) → CH_3_NH_3_PbBr_3_(aq) →CH_3_NH_2_(aq) + PbBr_2_(s) + HBr (aq)

**Fig. 2:**
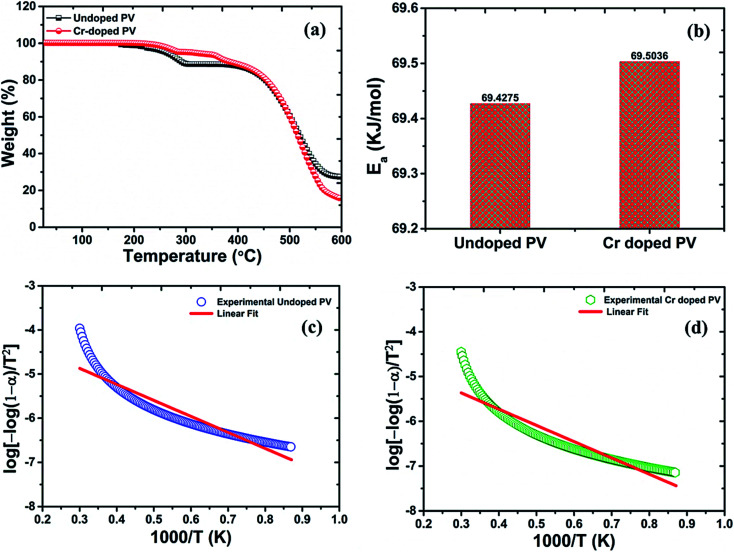
Thermogravimetric plots of (a) pristine PV (black line) and Cr-doped perovskite (red line), with (b) histogram comparisons of the calculated activation energy of pristine and Cr-doped perovskite nanocrystals, and a linear fit curve of (c) pristine PV (d) Cr-doped PV nanocrystals.

From the TGA spectrum (Fig. 2) of pristine MAPbBr_3_, a slight fraction of weight-loss was observed at ∼200 °C temperature (∼2% weight loss) due to decomposition of water molecules. In addition, the decomposition threshold of pristine perovskite nanocrystals at ∼262 °C (∼3% weight loss) is well suitable for perovskite nanocrystals for stable device applications. Moreover, the profile of pristine perovskite nanocrystals involves two major degradation steps including first degradation at ∼291 °C with a remaining weight of ∼88.58% from its original weight. In particular, pristine perovskite explores first degradation step of ∼11.42% weight loss corresponding to organic component methylammonium bromide (molar mass, *M*_MABr_  =  111.97 g mol^−1^) of as-grown pristine nanocrystals.^42–44^ In continuation, the major second degradation starts at ∼447 °C, which is attributed to degradation of PbBr_2_ (*M*_PbBr_2__ = 367.05 g mol^−1^) molecules, indicating that amine group is strongly bonded into hybrid perovskite crystal matrix. However, the weight-fraction (*α*) of pristine perovskite was also estimated to be about ∼16.8% at ∼300 °C temperature followed by major thermal degradation of ∼61.21% at ∼576 °C, which is attributed to melting of the PbBr_2_ precursor of perovskite nanocrystals.^45^ The precursor, lead acetate, itself might be removed at soft-bake low temperatures despite high risk of side-products in perovskite layer due to the high polarity. Thus, lead acetate might be converted into lead hydroxide or hydrate, PbO·H_2_O, to reduce lead quantity for perovskite conversion and retard the conversion-speed of precursors, as observed in EDX analysis.^44,45^ From Cr^3+^-doped perovskite nanocrystals, two small degradation steps have been observed at ∼260 °C with ∼4.96% mass-loss and ∼120 °C with ∼4.82% mass-loss, which are related to strong Cr bonding in perovskites and also found as small minima in percent derivative (Fig. 2a) curves due to removal of the Cr precursor, excessive HBr, and MABr molecules. Interestingly, Cr-doped MAPbBr_3_ nanocrystals demonstrate a decomposition temperature, *T*_d_, of ∼455 °C and a low weight loss of ∼8.57% compared to pristine perovskite (*T*_d_ = 447 °C) with a high mass destruction of ∼11.42% which indicate their high thermal stability under ambient conditions.^46^ In addition, major thermal degradation of ∼74.82% weight loss might be related to melting of the PbBr_2_ precursor with a weight residue ∼15.41% of Cr^3+^-doped perovskite nanocrystals. From TGA plots, Cr-doped perovskite nanocrystals provide a low weight fraction (*α*) of ∼5.7% at ∼300 °C temperature due to excellent doping, revealing high thermal stability compared to their pristine counterparts. Hence, the decomposition of perovskite nanocrystals is very low up to ∼282 °C (∼4% weight loss) and ∼363 °C (∼8% weight loss), which display further better thermal stability after Cr^3+^ doping for optoelectronic applications. The as-doped PVNCs provide huge incentives as potential candidates for long lifetime, high thermal stability, and optical and electronic applications.^47^

To support moisture and thermal stability, the DSC study was monitored for phase-transitions of doped perovskite nanocrystals. DSC plots (Fig. S1†) were recorded for phase conversion *via* heating and cooling scans of doped and pristine perovskite materials. The glass transition (*T*_g_) temperature of perovskite nanocrystals was reduced from ∼89 °C to ∼47 °C after Cr ion insertion. Furthermore, the melting transition (*T*_m_) temperature of doped perovskites might reduce after metal doping compared to their pristine perovskite materials resulting in conversion of phases. For pristine perovskites, MAPbBr_3_ nanocrystals, heating scan shows a small crystalline peak at ∼300 °C with a strong melting transition peak at ∼321 °C. Upon cooling, a glass transition peak was observed at ∼89 °C along with three crystalline peaks (Fig. S1a†) at ∼248, ∼252, and ∼260 °C, implying their phase reversibility for pristine perovskite. The absence of phase transitions for MAPbBr_3_ suggests low sensitivity towards thermal cycling, providing high tolerance to environmental retardation.^48^ After metal doping, the endothermic peaks exhibit significant enhancement in peak area, intensity, and reduction in particle size of perovskite nanocrystals. In DSC plots (Fig. S1b†), Cr-doped nanocrystals display a lower *T*_g_ peak at ∼46 °C followed by three small endothermic peaks at ∼241, ∼265 and ∼281 °C along with a strong crystalline peak at ∼310 °C in cooling cycle. During heating scan, a small crystalline peak at ∼281 °C was observed with a tiny melting peak at ∼302 °C along with a strong melting peak at ∼312 °C and a crystalline peak at ∼314 °C for Cr-doped perovskite nanocrystals.^49^

Under standard temperature conditions, the thermal decomposition kinetics of perovskite nanocrystals in a facile and simple approach has been established to investigate gradual mass-loss with temperature. Thus, activation energy values have been evaluated from TGA plots in model-free methods. The kinetic studies follow the rate equation as:

3 d(*α*)/d*t* = *kf*(*α*)

where *k* is the rate constant and *f*(*α*) the is reaction model function. The shape and structural stability of perovskite crystals might govern through composition and stoichiometry of precursors. The *k* value was evaluated using the Arrhenius equation:

4 *k* = *A* exp(−*E*/*RT*)

where *E*, *A*, *R*, and *T* are the activation energy (kJ mol^−1^), pre-exponential factor (min^−1^), standard gas constant (∼8.314 J K mol^−1^), and absolute temperature in Kelvin, respectively. Thus, the final equation is as follows:

5 d(*α*)/d*t* = *A* exp(−*E*/*RT*)*f*(*α*)

For the dynamic TGA process, the heating rate is expressed as follows:

6 *β* = d*T/*d*t*

Hence, the resulting equation can be found as follows:

7 d(*α*)/d*T* = (*A*/*β*) exp(−*E*/*RT*)*f*(*α*)

Thus, by using these equations, we can theoretically calculate the kinetic parameters from TGA analytical data of perovskite nanocrystals. The activation-energy can be evaluated by the slope of ln (*σT*) against 1/*T* plots (Fig. 2c) of perovskite and was estimated as ∼69.4275 kJ mol^−1^ for pristine perovskite nanocrystals.^50^ Due to Cr^3+^ doping, the estimated activation energy was extracted as ∼69.5036 kJ mol^−1^ (Fig. 2d) for Cr-doped perovskite nanocrystals using above-mentioned linear-fit method, which clearly indicates the increment of ∼76.10 J mol^−1^ in activation energy after doping compared to pristine perovskite nanocrystals, as shown in the histogram profile in Fig. 2b. Hence, it might be easily concluded that as-grown rod-like Cr-doped perovskite nanocrystals require more energy to surpass their threshold energy-barrier, resulting in overall improved thermal stability.^51^ After Cr^3+^ doping, the combine effect of small-sized nanocrystals, efficient Cr insertion and partial replacement of lead cations is responsible for stable doped perovskite nanocrystals.

### Morphological properties of perovskite nanocrystals

**Fig. 3:**
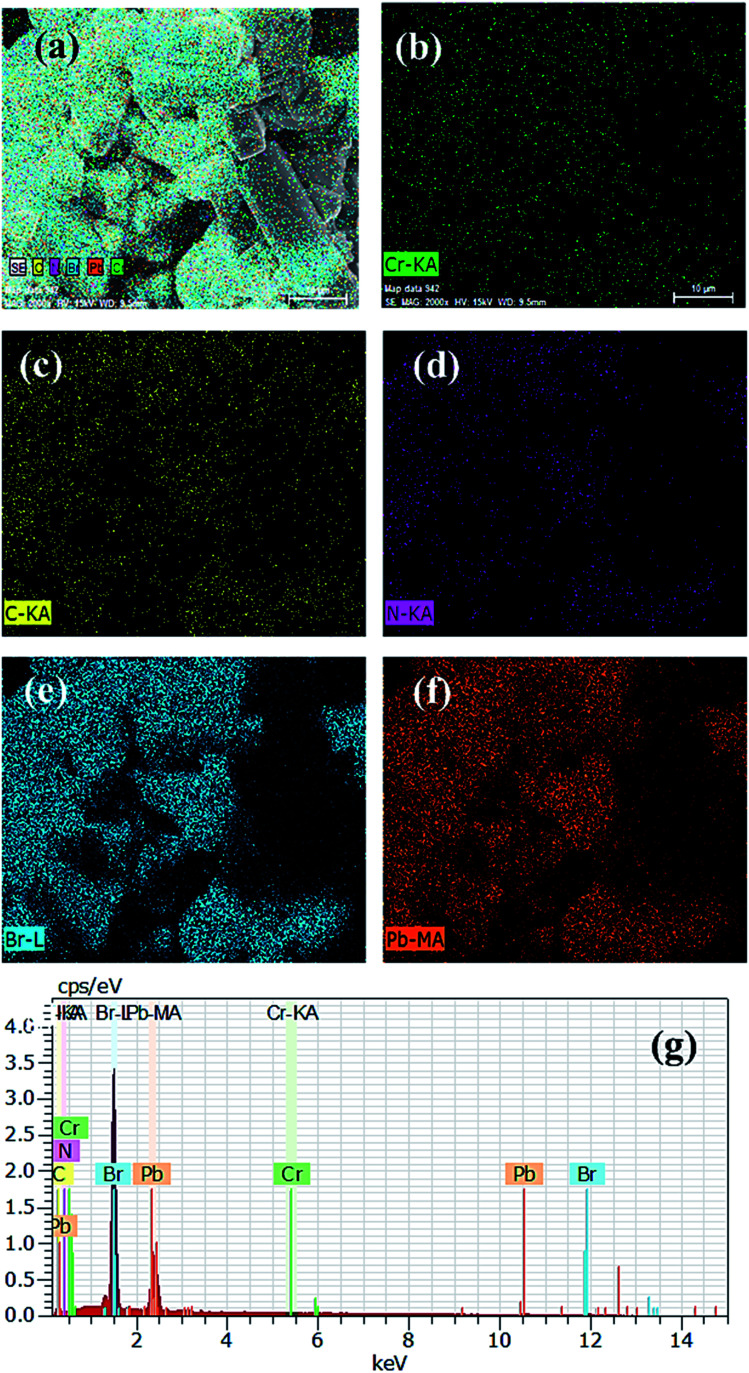
(a) FESEM images, (b) Cr element, (c) C element, (d) N element, (e) Br element, (f) Pb element, and (g) energy-dispersive index X-ray spectrum (EDX) of Cr-doped perovskite nanocrystals.

From the FESEM images, it is clear that pristine PVNCs contain hexagonal cubic crystals (Fig. S2†) with the size in the range of ∼200–500 nm developed from a low-temperature method. Due to its strong oxidizing properties, Cr cations were employed as efficient dopants to partially replace Pb cations of perovskite, CH_3_NH_3_PbBr_3_ nanocrystals in ambient air. Owing to small size of Cr^3+^ cations, the as-grown doped perovskite nanocrystals possess a length of <50 nm and a width of <10 nm as a rod-like (Fig. 3a) nanocrystal structural morphology. Interestingly, pristine perovskite nanocrystals showed aggregation of a large number of cubic perovskite nanocrystals, while Cr doping produced a rod-like nanocrystal structure at low temperatures.^52^ Furthermore, crystallinity slightly reduces after Cr^3+^ doping, but retains its cubic crystal phase structure with no trace of other phase peaks in doped nanocrystals. The elemental mapping images exhibited that Cr^3+^ cations were homogenously distributed in perovskite grains along with other elements. Furthermore, SEM mapping images of pristine perovskite (Fig. S2e–g†) and doped perovskite (Fig. 3c–g) nanocrystals showed smooth and consistent distribution of Cr, Pb, and Br atoms. Normally, energy orbitals of Cr^3+^ cations are greatly affected by their surrounding coordination environments designed by the presence of coordinating ligands, which resulted in tunable optical properties due to presence of weak octahedral crystal field ligand, Cl^−^ anions.^53^ In EDX analysis, the atomic ratio of basic elements Pb : Br exhibited a ratio of 1 : 3.10 (Fig. S2a†), which follows with exact stoichiometry and homogenous elemental distribution (Fig. S2b–e†) of pristine perovskite MAPbBr_3_ nanocrystals. In addition, transmission electron microscopic (TEM) images (Fig. S3a–e†) also revealed a rod-like structure of Cr-doped CH_3_NH_3_PbBr_3_ nanocrystals having their amorphous nature and significant contraction of crystal structure. Thus, rod-like Cr-doped nanocrystals display excellent FFT patterns and lattice spacings for slight phase conversion after doping, while perovskite nanocrystals provide a Pb : Br atomic ratio (Fig. 3g) of 1 : 3.49 after doping, which might be due to slight excess of hydrobromic acid addition during growth of perovskite nanocrystals. Owing to odd stoichiometry of components or excess of halide or incomplete conversion of precursor into perovskite, thin films will have minute amounts of one or both precursors in their ionic form, Pb^+2^ cations or Br^−^ anions.^54^ Thus, doped perovskite nanocrystals might be evaluated with an optimized concentration of ∼13% of Cr^3+^ cations to replace Pb^2+^ cations in ambient atmosphere. Importantly, TEM and EDX mapping analyses were applied to evaluate uniform distribution of Cr^3+^ cation distribution inside perovskite grains. Owing to small size of cations, insertion of Cr^3+^ cations has various interactions with bromide ions to decrease ion-migration barriers, resulting in significant contraction of perovskite nanocrystal lattice with ultra-pure crystal grains.

### Degree of crystallinity and XRD analysis

**Fig. 4:**
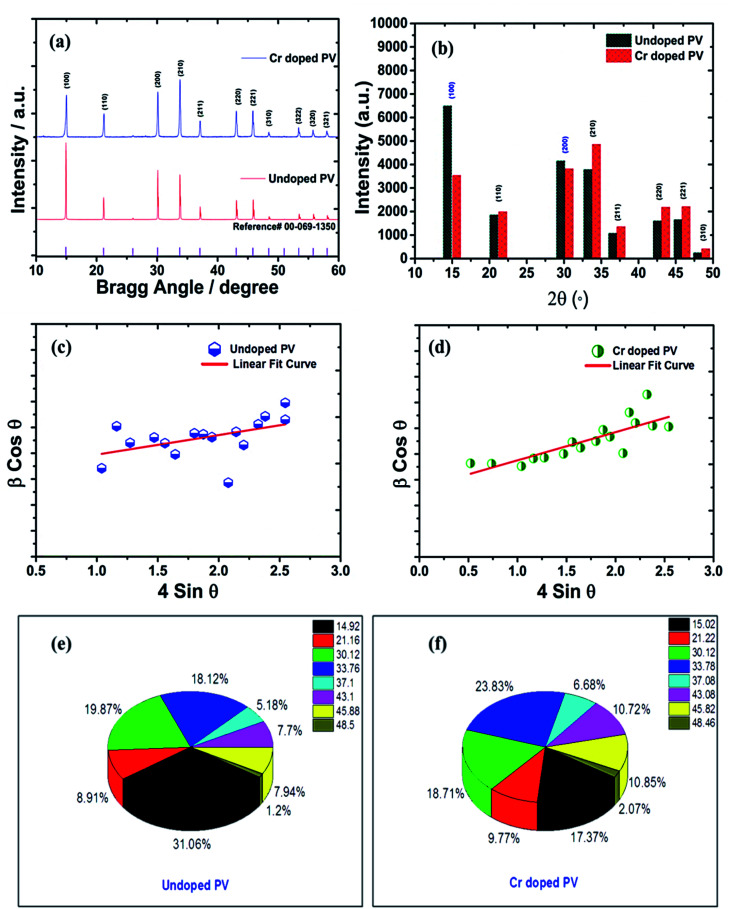
(a) Comparative XRD spectra of pristine and Cr-doped perovskite nanocrystals with reference (reference card number; 00-069-1350) spectra (b) histogram profile, and W–H plots of (c) pristine, (d) Cr-doped perovskite nanocrystals, and π-chart presentation of various XRD peaks of (e) pristine, and (f) Cr-doped perovskite nanocrystals.

The crystal lattice of perovskite nanocrystals was estimated by XRD (Fig. 4) analysis for perovskite nanomaterials. The pristine CH_3_NH_3_PbBr_3_ perovskite nanocrystals explore high-intensity crystalline diffraction peaks at Bragg's angles of ∼14.92°, ∼21.22°, ∼30.12°, ∼33.76°, ∼37.10°, ∼43.10°, ∼45.88°, ∼53.48°, and ∼64.76°, which are attributed to the (100), (110), (200), (210) (211), (220) (221), (222) and (400) space groups, respectively. The XRD peak values are well matched with the spectrum of high-crystalline pristine cubic (ICSD card no. 00-069-1350) perovskite phase.^55^ The XRD peaks at ∼14.92° and ∼30.12° of the (100) and (200) space groups (Fig. 4a) display an intensity decrease, while all other XRD peaks have shown a slight increment in peak intensity after Cr insertion in perovskite nanocrystals. In particular, peak at (100) with ∼14.92° moves to ∼15.01° with a ∼45.45% lowering of peak intensity (histogram profile, Fig. 4b) with a decreasing FWHM value of ∼0.19° than ∼0.25° of pristine CH_3_NH_3_PbBr_3_ nanocrystals. However, XRD peak at ∼30.12° remains at similar Bragg angles with a significant reduction of ∼8.18% of peak intensity after Cr^3+^ doping. In addition, there is no trace of presence of PbBr_2_ peaks in XRD spectra, which further evidenced to ultrapure and excellent crystalline nanocrystals. The Cr^3+^ cations (2.5 wt%, 5.0 wt% and 7.5 wt%) were applied for doping perovskites, and XRD plots (Fig. S4a†) report a significant reduction in peak intensity for ∼2.5 wt% doping, which further decrease with increase (Fig. S4b†) in the concentration of Cr^3+^ cations (∼5.0 wt%) along with a slight increment in the Bragg angle values. After efficient metal doping, the nucleation growth of perovskite crystals might control crystal assembly (Fig. S4c and d†), which affects physico-chemical properties of perovskite nanocrystals.^56^ The Cr^3+^ cation doping might influence crystal grain size, and activation energy of perovskite crystals results in various crystal defects. On the other side, Cr^3+^ cations affect crystal lattice of pristine hybrid perovskites, which leads to cubic crystal phase retention after doping, while peak broadening indicates that crystal refinement with grain size supports lattice-strain features of perovskite nanocrystals. The grain size of perovskite crystals can be achieved using the Debye–Scherrer formula:8
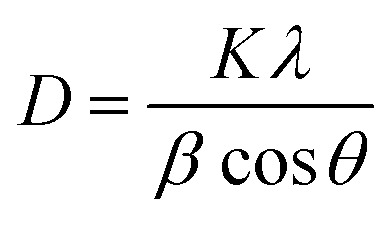
where *D*, *K*, *β*, *θ* and *λ* are the crystallite size, shape factor (0.94), peak broadening at FWHM value, Bragg angle, and X-ray wavelength of Cu kα radiation, respectively. Additionally, crystallinity (Table 1) of perovskite crystals can be obtained as follows:9
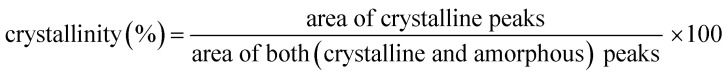


**Table 1:** Thermal stability parameters of PVNCs

HOME PVNCs		*T*_d_ (°C)	*T*_g_ (°C)	*T*_c_ (°C)	*T*_m_ (°C)	*α*_300_ (%)	Mass_@300_ (%)	*E*_a_ (kJ mol^−1^)
Pristine PV	174, 285, 516		89	248, 252, 260	321	16.8	88.59	69.4275
Cr doped PV	274, 364, 522		47	241, 265, 310	312	5.67	95.27	69.5036

Furthermore, Cr^3+^ cation-doped perovskite nanocrystals lead to low crystallinity of ∼67% than that of ∼75% for pristine perovskite nanocrystals. Notably, the efficient and partial replacement of large sized, Pb^2+^ cations (an ionic radius 119 pm) has been done by relatively small-sized Cr^3+^ cations (ionic radius 62 pm) resulting in substantial crystal-size contraction from ∼193 nm to ∼48 nm of rod-like nanocrystals after doping at low temperatures.^57^

### Lattice strain engineering and W–H analysis

The hybrid perovskite nanocrystals were analyzed by an integral method using W–H plots (Fig. 4c and d) to determine their crystal size and strain properties, which induce the peak broadening area. The W–H plot exhibits significant differences in lattice-strains due to efficient doping, which creates crystal imperfections and distortions at grain-boundaries for pristine and Cr^3+^-doped perovskite nanocrystals. Hence, the crystal imperfection and distortion in terms of crystal lattice strain (*ε*) were calculated using the following relation:10
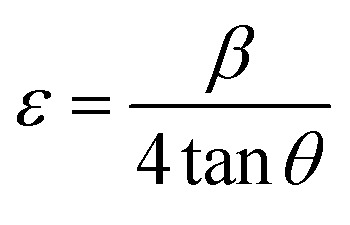


The line broadening of XRD peaks might be affected by size and lattice-strain of perovskite nanocrystals. The lowering of intermolecular distance (*d*) of XRD peaks induces peak broadening of high-crystalline materials.11
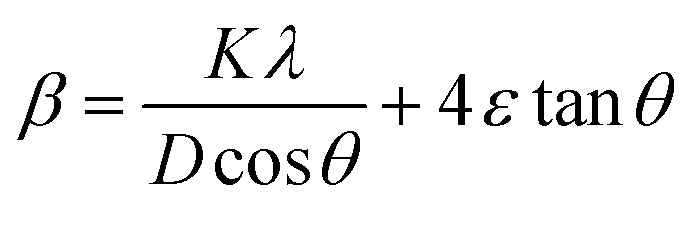


Furthermore, Cr^3+^-doped PVNCs exhibit a high crystal lattice-strain of ∼4.52 × 10^−4^ compared to ∼1.18 × 10^−4^ for pristine PVNCs evidenced induction of Cr^3+^ cations to stabilize the perovskite crystal lattice. Hence, lattice-strain increases after doping by atomic reorganization in crystals which significantly reduce crystal size with phase-retention of crystalline cubic geometry of pristine perovskite.^58^ The dislocation density (*δ*) of crystalline materials can be obtained from the Williamson and Smallman relation:

12 *D*^2^*δ* = 1

The *δ* value of crystalline materials depends on crystallite size of perovskite nanocrystals. Thus pristine perovskite nanocrystals exhibit a dislocation density of ∼2.675 × 10^13^ lines per m^−2^. The size of pristine nanocrystal (∼193 nm) significantly reduced up to ∼48 nm after doping with Cr^3+^ cations. Hence, the *δ* value has been drastically improved up to ∼4.24 × 10^14^ lines per m^−2^ for Cr-doped perovskite nanocrystals. Thus, various intensity percentages have been depicted by pi-charts (Fig. 4e and f) for pristine and Cr^3+^-doped perovskite nanocrystals, while peak of crystal phases (100) and (200) with ∼31.06% and ∼19.87% have been reduced to ∼17.37% and ∼18.71% after doping. In addition, the (220) crystal phase has explored an increment of ∼23.83% in Cr^3+^-doped nanocrystals from ∼18.12% of pristine perovskite nanocrystals.^59^

### Stoichiometry of perovskite nanocrystals and substitutional doping engineering

In a crystal lattice, size of foreign materials is a decisive factor for its insertion *via* doping-engineering. Henceforth, efficient incorporation of metal cations or replacement of any metal cations has provided very unique, useful and interesting features into doped nanomaterials.^60^ Owing to small ionic radius, various metal ions provide a great probability to insert into hybrid perovskite lattice in a relatively simple and facile approach, which might significantly improves their stability. However, crystal defect chemistry of perovskite nanomaterials has been drawing tremendous attention due to their robust, facile, cost-effective ways of handling doping techniques. Of course, partial replacement of Pb^2+^ cations might reduce its toxicity, resulting in production of eco-friendly perovskite nanocrystalline materials. The oxidation state of dopant chromium cation displays a stable electronic configuration in Cr^3+^ high-spin (HS, t^3^_2g_ e^0^_g_) state (compared to Cr^2+^, HS, t^3^_2g_ e^1^_g_) octahedral field, which provides a low-cost, green and eco-friendly synthesis method at low temperatures.^61^

In doped MAPbBr_3_ crystal lattices, the atomic positions might change in cubic crystal structure due to crystal phase conversion. From XRD spectra and EDX analysis, it can be revealed that transition metal (Cr^3+^, HS) cations inserted into perovskite crystal lattice partially replace Pb^2+^ cations. The theoretical estimation of Goldschmidt's tolerance factor (*t*) and octahedral factor (*μ*) generally applied for a benchmark of stability, phase and geometry of perovskite-type nanomaterials. In addition, *t* determines the degree of crystal deformation after metal doping to induce structural stability and might be calculated as follows:13
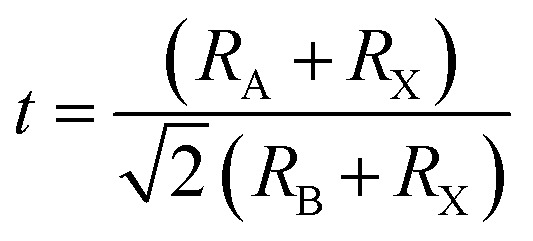
where ionic radii of A cation, B cations and Br^−^ anion belong to *R*_A_, *R*_B_, and *R*_X_, respectively. Thus, effective ionic radii (*r*_effective_) of metal-doped perovskites were obtained after lead substitution by Cr^3+^ cations *via* chemical doping. These ionic radii are taken in a six-coordinate halide system, which translated directly as the bond-length of octahedron of 3D perovskite nanomaterials. Thus obtained tolerance factor (*t*) of nanomaterials should be 0.81 to 1.10 range to get ideal cubic crystal structure of perovskite-type materials.^62^ Thus, metal cation substitution of perovskite materials might enhance their structural stability of doped perovskite nanocrystal effective doping. Meanwhile, the B–X bond length plays a crucial role in band gap energy, which decreases slightly after Cr^3+^ cation doping. Hence, effective radii of Cr^3+^ cation-doped perovskite nanocrystals formulated as CH_3_NH_3_Cr_0.12_Pb_0.88_Br_3_ which might be precisely calculated as follows:

14 *R*_A(effective)_ = *xR*_A′_ + (1 − *x*)*R*_A′′_

15 *R*_B(effective)_ = *xR*_B′_ + (1 − *x*)*R*_B′_

where the effective ionic radius of cations A and B is *R*_A(effective)_ and *R*_B(effective)_, respectively. Then, *x* is doping percentage of Cr^3+^ cations, and *R*_B′_ and *R*_B′′_ belong to ionic radius of R_Cr_^3+^ cations and Pb^2+^ cations, respectively. Hence, effective *t* of doped perovskite nanocrystals might be calculated as follows:16
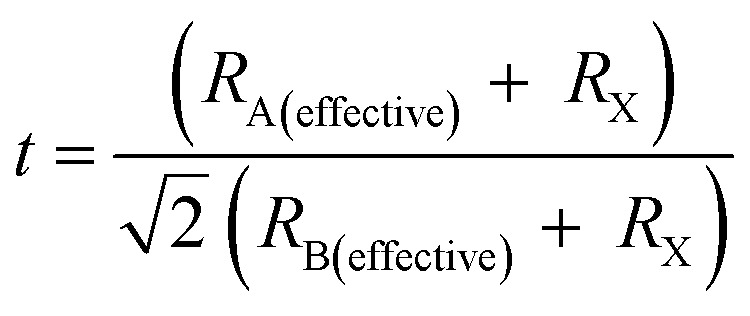


Hence, effective *t* value follows stability in 0.8 < *t* < 1 range related to cubic crystal phase after metal-doping with no change in crystal phase of perovskite materials. Hence, trivalent Cr^3+^ cations might insert into perovskite crystals due to their small size with high-polarity Cr–Br bonds compared to Pb–Br bonds, resulting in high stability of doped perovskite nanocrystals. Hence, the general formula of Cr-based perovskite nanocrystals is postulated as CH_3_NH_3_Cr_*x*_Pb_1−*x*_Br_3_ to evaluate the *t* value (Table 1) of ∼0.95 compared to that of pristine of ∼0.93, which belongs to specified range to retain their cubic crystal geometry of pristine perovskite nanocrystals.^63^

The *μ* is a dimensionless number obtained using the relation including ionic radii of metal cations:

17 *μ* = *R*_B_/*R*_X_

Thus, the cubic structure can be estimated by numerical value of *μ* from 0.44 to 0.89 of halide perovskite materials. In this work, *μ* was calculated to be ∼0.61 for pristine perovskite nanocrystals, which reduces to ∼0.57 after insertion of Cr cations into PVNCs and follow the specified range resulting in a stable structural geometry with slight contraction but retention of cubic crystal phase. The XRD plots (Fig. S5†) of Cr-doped perovskites display excellent phase-stability with high-intensity peaks up to ∼150 °C. However, a further increase in temperature (∼200 °C) slightly lowers intensity of peaks with retention of cubic phase of PVNCs.

### Optical properties and band gap engineering

**Fig. 5:**
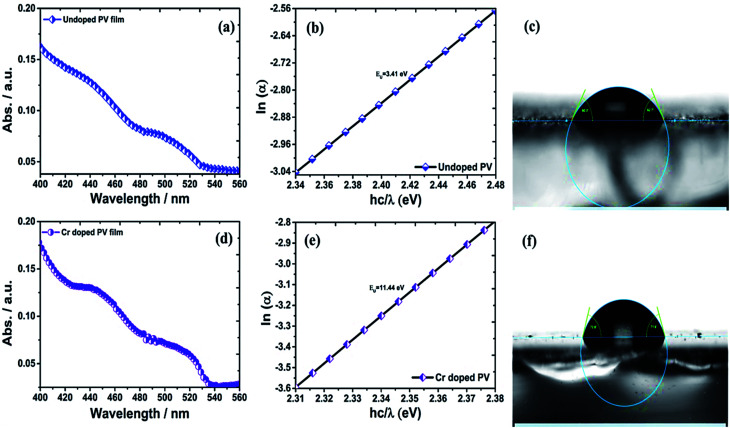
(a) UV absorption spectra, (b) Urbach energy plot, and (c) contact angle analysis of pristine perovskite nanocrystals, and (d) UV absorption spectra plot, (e) Urbach energy plot, and (f) contact angle analysis of Cr-doped perovskite nanocrystals.

The ITO-based thin films of pristine and doped CH_3_NH_3_PbBr_3_ (Fig. 5) revealed excellent optical properties of PVNCs in absorption spectra. The energy band gap of perovskite nanocrystals was evaluated from corresponding Tauc plots (Fig. S6†) of UV spectra in terms of valence and conduction (VB and CB) bands. When an electron of VB excited into CB after light absorption, the band gap (*E*_g_) energy can be calculated by using following equation:

18 *α*h*ν* = (h*ν* − *E*_g_)^2^

where *α*, h*ν*, and *A* are absorption coefficient, energy, and constant, respectively. *E*_g_ can be evaluated from following relation:19
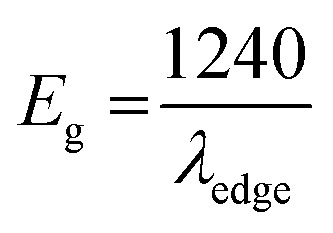


In continuation, lead bromide is converted into CH_3_NH_3_PbBr_3_ by a color change of thin film from yellow to orange color of perovskites after annealing at 100 °C for 10 min. From UV plots (Fig. 5a and d), the onset value of absorption was calculated as ∼530 nm (Table 2) for pristine CH_3_NH_3_PbBr_3_ films corresponding to an energy band gap of ∼2.33 eV using Tauc plots (Fig. S6a and b†), revealing CH_3_NH_3_PbBr_3_ crystal formation after 10 min annealing.^64^ Additionally, Cr-doped perovskite thin films displayed an onset value of ∼535 nm of absorption wavelength, resulting in ∼2.31 eV band gap energy. Furthermore, metal-alloy might form thin films due to metal cation insertion in perovskite crystal lattice, which boosts transfer of electronic charges in perovskite nanocrystals.

**Table 2:** Structural analysis of HOME PVNCs from XRD spectral plots

HOME PVNCs	Crystallite size (nm)	FWHM (°)	Crystallinity (%)	Tolerance factor (*t*)	Octahedral factor (*μ*)	Dislocation-density (*δ*, m^−2^) ×10^14^	Strain (*ε*) × 10^−4^
Pristine PV	193	0.25	75	0.93	0.61	0.268	1.18
Cr doped PV	48	0.19	67	0.95	0.57	4.24	4.52

### Urbach energy (*E*_U_) estimation

In thin films, the Urbach energy designates disorder of phonon states, which is governed by structural disorder and stoichiometric defects at surface. The Urbach energy has been observed as absorption of photons in a large number of amorphous materials, which is found below absorption band edge of compounds. The Urbach energy (Fig. 5) is considered as an important parameter with crystallinity of nanomaterials to control their order of crystalline structures. Generally, crystalline compounds might trap electrons in excited states from defects to protect electronic transitions towards conduction bands, which are considered as tail of absorption or Urbach tail.^65^ Furthermore, energy related to this absorption tail is called the Urbach energy (E_U_, Table 3) and estimated as exponential of absorption coefficient (*α*) using the following equation:20
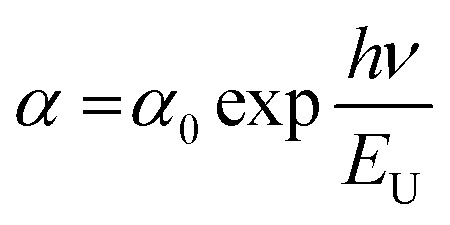
where *α*_0_ is a constant. *E*_U_ slightly depends on the localized states of low crystalline or amorphous materials and temperature. The logarithm of above-mentioned relation can be modified as follows:21
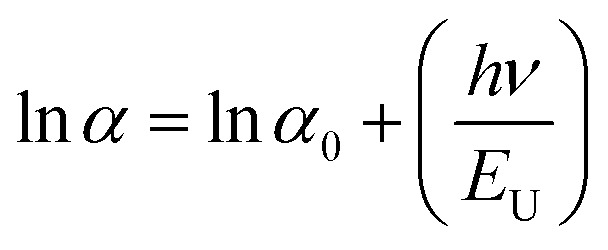


**Table 3:** Optical parameters of perovskites extracted from UV-visible and PL spectral analysis

Perovskite nanocrystals	Abs. edge (*λ*_edge_)^a^ (nm)	Energy band gap^b^ (eV)	Urbach energy (*E*_U_)^c^ (eV)	Contact angle^d^ (°)
Pristine PV	530	2.33	3.41	61.5
Cr doped PV	535	2.31	11.44	72.2

a Estimated from the absorption spectra of PV films.

b Obtained from the Tauc plots of perovskite thin films.

c Estimated from the ln (*α*) *vs.* energy band gap plots.

d Obtained from the contact angle measurement of PV thin films against water drops.

For doped perovskites, the E_U_ value (Fig. 5b) exhibits a significant enhancement of ∼11.44 eV compared to ∼3.41 eV of pristine perovskite (Fig. 5e) nanocrystals. The significant increment in Urbach energy causes a large random movement of atoms in crystal stacked structure of low crystallinity after Cr ion doping, which induces a vibrational energy resulting in high multiplicity. For doped perovskite nanocrystals, improved *E*_U_ results in high energetic disorder of band edges in structurally disordered semiconductors.

### Hydrophobicity of Cr-doped perovskite nanocrystals

In addition, contact angle measurements (Fig. 5) have been applied against water drops to assess hydrophobicity of as-grown perovskite nanocrystals with or without doping. It was found that thin-film surface of Cr-doped perovskite nanocrystals produced improved contact angle and high hydrophobicity, resulting in an increased stability after doping.^66,67^ Moreover, the moisture stability and water resistivity of perovskite thin films have been applied to find stability against water for pristine a contact angle of ∼61.5° (Fig. 5c) in ambient atmosphere. Furthermore, contact angle (Table 3) of Cr^3+^-doped nanocrystal films have significantly increased up to ∼72.2° (Fig. 5f), resulting in a high hydrophobic nature and excellent water stability under ambient conditions. Thus, it was concluded that small-sized cations easily and efficiently replaced toxic Pb elements to grow less toxic, excellently hydrophobic and highly stable perovskite nanocrystals for future applications.^68^

### XPS analysis

**Fig. 6:**
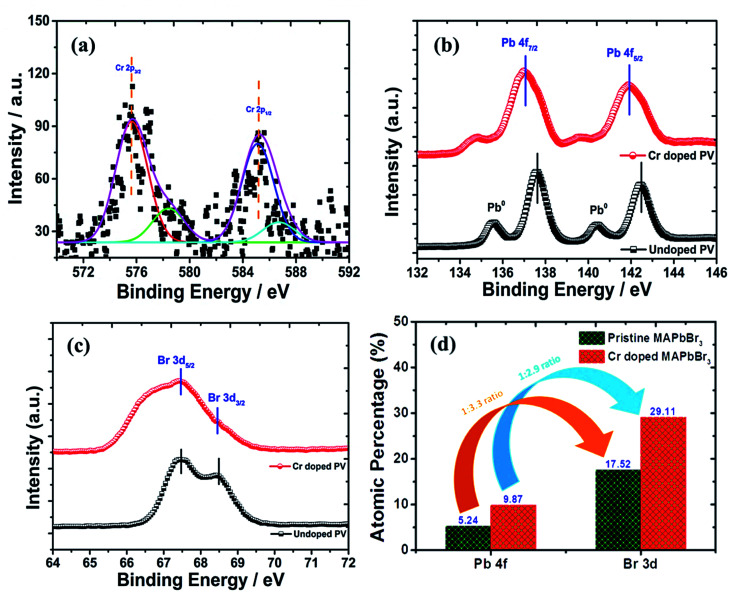
XPS spectral plots of (a) Cr 2p spectrum, (b) Pb 4f spectra, (c) Br 3d spectrum and (d) histogram profile of the relative change in Pb : Br ratios of Cr-doped perovskite nanocrystals.

The bonding and structural modifications of perovskite nanocrystals were measured using XPS plots (Fig. 6) in terms of binding energy estimation. Additionally, fitted XPS plots of Cr 2p_3/2_ and Cr 2p_1/2_ display (Fig. 6a) ∼576 eV and ∼586 eV binding energy for Cr^+3^ ionic states, respectively. However, these major peaks were convoluted further into two binding energy peaks each at ∼575.63, and ∼578.45 eV assigned to Cr 2p_3/2_ along with ∼585.22 and ∼586.76 eV binding energy convoluted peaks for Cr 2p_1/2_ of variable oxidation states.

In pristine perovskite nanocrystals, there were two characteristic, sharp and high-intensity elemental peaks of Pb^+2^ cations (Fig. 6b) found at ∼137.61 eV binding energy for Pb 4f_7/2_ and ∼142.47 eV for Pb 4f_5/2_, resulting in ∼4.86 eV of spin–orbit splitting (Δ*E*) energy with some traces of lead metallic (Pb^0^) peaks. In addition, the peak intensity and binding energy of respective lead metal peaks were significantly lowered due to enhanced perovskite conversion in Cr-doped perovskite nanocrystals with no traces of lead oxide components.^69^ However, a peak binding energy of Pb^+2^ cations was lowered at ∼137.01 eV and ∼141.88 eV for Pb 4f_7/2_ and Pb 4f_5/2_, respectively, to produce enhanced Δ*E* of ∼4.88 eV after insertion of Cr^+2^ cations into perovskite nanocrystals. Thus, the binding energy has been reduced by ∼0.60 eV and ∼0.41 eV for Pb 4f_7/2_ and Pb 4f_5/2_ peaks, respectively after Cr^3+^ doping. It was demonstrated that PbBr_2_ formation after HBr insertion and its conversion into hybrid perovskite, CH_3_NH_3_PbBr_3_, occurred in a molecular exchange reaction between CH_3_NH_3_Br and lead acetate precursor with addition of ethanol as an antisolvent. In pristine perovskites, two Br 3d peaks (Fig. 6c) were observed at ∼67.48 and ∼68.45 eV binding energies attributed to Br 3d_5/2_ and Br 3d_3/2_ peaks, respectively. Thus, Br 3d peaks slightly shift to a lower binding energy, which is probably due to formation of a new intermediate Pb–Br–Cr complex and precursor lead acetate bonds along with Pb^2+^ cations with Br^−^ anion interaction bonds. Hence, the Br 3d spectrum exhibits a decrease in binding energy of ∼0.81 eV for Br 3d_5/2_ and ∼1.01 eV for Br 3d_3/2_ peaks after doping of Cr in perovskite nanocrystals.^70,71^ From the XPS analysis, perovskite nanocrystals display a Pb/Br ratio (Fig. 6d) of 1/3.3 for pristine, which is matched with the stoichiometry of perovskites. However, the Pb/Br ratio has been modified as 1/2.9 after Cr doping, which might be due to partial substitution of Pb, resulting in the reduction rate of perovskite conversion.

Furthermore, the peaks at ∼285 eV, and ∼400 eV binding energies were assigned to C 1s, and N 1s elements, respectively. The histogram profile of various elements of doped perovskite nanocrystals (Fig. S7†) provides types of bonding interaction possibilities after doping. In doped perovskite nanocrystals, the C 1s spectrum (Fig. S7a†) showed a broad asymmetric peak centered at ∼283 eV, which is assigned to methyl carbons in CH_3_NH_3_PbBr_3_ nanocrystals.^72,73^ The C 1s broad peak convolutes into two peaks at ∼283.09, and ∼284.19 eV binding energies. Among them, a broad convoluted peak of high intensity at a binding energy of ∼283.09 eV is related to sp^3^ C–C bonds of methylamine molecules. However, the convoluted peak of low intensity at a binding energy of ∼284.03 eV represents C–N bonds of Cr-doped perovskite nanocrystals. In continuation, the N 1s spectrum (Fig. S7b†) explores a peak of binding energy ∼400 eV, which majorly represents C–N bonds constructed with methylammonium bromide molecules and Pb precursor materials. The highest intensity peak of N 1s XPS spectra was attributed for sp^3^ C–N bond of CH_3_NH_3_ at ∼400.77 eV along with a peak at ∼399.76 eV binding energy for carbon-to-nitrogen (C–N) bond of nanocrystals. Along with this, the emergence of a low intensity peak at ∼401.67 eV corresponding to C–NH_3_ bond reveals a weak hydrogen-bonding possibility in MA^+^ (CH_3_NH_3_^+^) cations of perovskite nanocrystals. The variation in metal concentration histogram profiles (Figure S7c†) is presented for perovskite nanocrystals before and after Cr doping under ambient conditions.^74,75^

### Raman analysis of Cr-doped perovskite nanocrystals

**Fig. 7:**
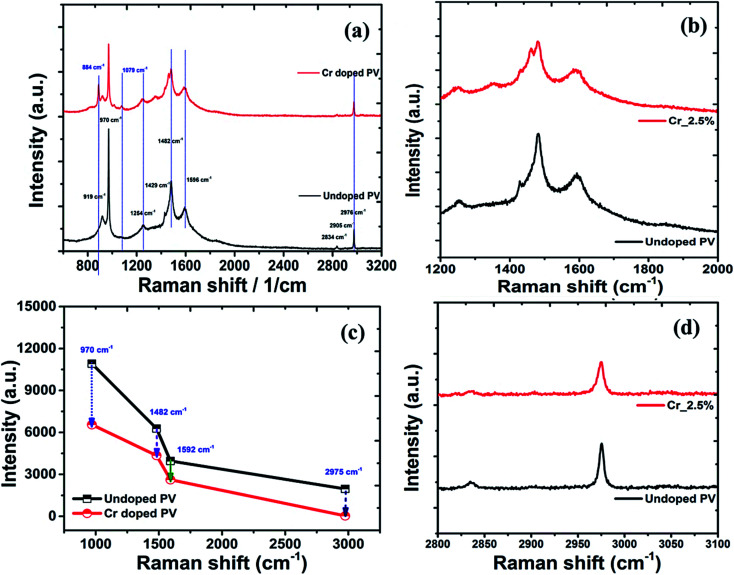
(a) Raman spectral plots of perovskite nanocrystals, with high-resolution spectra in the low-frequency region, (b) related C–N bands, (c) C–C bands, (d) C–H band spectra plot perovskite nanocrystals.

The doping effects on pristine MAPbBr_3_ nanocrystals were studied under laser radiation using *in situ* Raman spectra (Fig. 7) of MAPbBr_3_ samples. For degradation and doping mechanisms, it is postulated that a perovskite nanomaterial faces simultaneous breaking and formation of various chemical bonds, which can be reflected by Raman spectroscopic analysis.^76^ In Raman spectra, pristine perovskite nanocrystals (Fig. 7a) display major and high-intensity peaks at wavenumber frequencies of ∼919, ∼970, ∼1254, ∼1429, ∼1482, ∼1593, ∼2834, and ∼2976 cm^−1^. The sharp and intense bands at ∼970 cm^−1^ are assigned to the C–N stretching vibration, while that at ∼1482 cm^−1^ is ascribed to asymmetric bending vibration of –NH_3_^+^ cations, respectively. After Cr doping, these peaks showed significant red-shift compared to pristine perovskite samples, indicating strong bonding interactions in a hybrid crystal framework. In addition, rocking vibration modes at ∼922, ∼1254, and ∼1429 cm^−1^ show a slight blue shift on doping.^77^ Among them, a rocking vibration mode at ∼1254 cm^−1^ and a twisting vibration mode (–NH_3_^+^) at ∼1593 cm^−1^ in crystal MAPbBr_3_ are quite weak and slightly red-shifted for Cr-doped perovskite nanocrystals (Fig. 7b), suggesting improved bonding interactions for enhanced stability. The Cr doped perovskite nanocrystals show strong peaks at ∼921, ∼1411, ∼1461 and ∼1568 cm^−1^, which are attributed to CH_3_–NH_3_^+^ rocking, C–N stretching of CH_3_NH_3_^+^, symmetric and asymmetric NH_3_^+^ bending, and twisting vibration of NH_3_^+^ cation frequency modes, respectively.^78^ In continuation, four major peaks as ∼970, ∼1482, ∼1592, and ∼2976 cm^−1^ explored a relative decrease in intensity (Fig. 7c) of ∼39.9%, ∼30.7%, ∼33.8% and ∼26.7%, respectively after Cr doping. The interaction capability of perovskite nanocrystals was further revealed by inspecting vibration band shifting of C–N, NH_3_^+^ and CH_3_ stretching of CH_3_NH_3_^+^ after doping. Owing to lone-pair electrons, the interaction of CH_3_NH_3_^+^ cations with PbBr_3_^−^ anions forms hydrogen bonding as a N^+^–H----Br complex in octahedral framework, resulting in blue-shifts of C–N stretching bands. In addition, the asymmetric stretching band of CH_3_ was situated at ∼2976 cm^−1^ with a symmetric stretching band of N–H at ∼2836 cm^−1^ in high-frequency region. In addition, a broad symmetric stretching band of –NH_3_^+^ cations was red-shifted to ∼3036 cm^−1^ for Cr-doped perovskite (Fig. 7d) compared to ∼3045 cm^−1^ of pristine perovskite nanocrystals.^79^

## Conclusions

In summary, we have developed stable Cr^3+^-doped CH_3_NH_3_PbBr_3_ nanocrystals by an eco-friendly method at low temperatures using ethanol as antisolvent. The Cr^3+^ cations were doped into a CH_3_NH_3_PbBr_3_ crystal structure to tune their physical, thermal and electrical properties. After doping, PVNCs provided a reduced crystallite size of ∼48 nm and a FWHM of ∼0.19° for Cr^3+^ cation–doped perovskites compared to pristine perovskite crystallite size (∼193 nm) due to small size of cations used for insertion. Thus obtained Cr^3+^-doped PVNCs have negligible effect on the crystalline cubic phase (ICSD No. 00-069-1350) of pristine perovskite with an enhanced *t* ∼0.95 with *μ* of ∼0.58 for doped perovskite nanomaterials. The Cr^3+^ doped nanocrystal-based thin films display high hydrophobicity against water with high contact angle of ∼72° compared to ∼63° for their pristine counterpart. Owing to high thermal and humid stability, such PVNCs might be promising light-harvesting materials for optoelectronic applications in future.

## Conflicts of interest

The authors declare no competing financial interests.

## Supplementary Material

NA-004-D2NA00053A-s001
