# Supplementary material for: Insertion of metal cations into hybrid organometallic halide perovskite nanocrystals for enhanced stability: eco-friendly synthesis, lattice strain engineering, and defect chemistry studies
Source: Nanoscale Adv. 2022 May 12;4(12):2729–43. doi: 10.1039/d2na00053a (PMC9419879; doi:10.1039/d2na00053a)
Supplement: NA-004-D2NA00053A-s001 [file NA-004-D2NA00053A-s001.pdf]

Electronic Supplementary Material (ESI) for Nanoscale Advances.
This journal is © The Royal Society of Chemistry 2022

Supporting Information

Inserting Metal Cation in Hybrid Organometallic Halide Perovskite Nanocrystals for Enhanced Stability: Eco-friendly Synthesis, Lattice Strain Engineering, and Defect Chemistry Studies

Mohammed Nazim[a,*], Aftab Aslam Parwaz Khan[c,*], Firoz Khan[d], Sung Ki Cho[a,b] and Rafiq Ahmad[e,*]

[a]Department of Chemical Engineering, Kumoh National Institute of Technology, 61 Daehak-ro, Gumi-si, Gyeongbuk-do 39177, Republic of Korea

[b]Department of Energy Engineering Convergence, Kumoh National Institute of Technology, 61 Daehak-ro, Gumi-si, Gyeongsangbuk-do 39177, Republic of Korea

[c]Chemistry Department, Faculty of Science, King Abdulaziz University, P. O. Box 80203, Jeddah 21589, Saudi Arabia

[d]Interdisciplinary Research Center for Renewable Energy and Power System (IRC-REPS), King Fahd University of Petroleum & Minerals (KFUPM), Dhahran, 31261, Saudi Arabia

[e]Centre for Nanoscience and Nanotechnology, Jamia Millia Islamia, New Delhi-110025, India

*Email: nazimopv@gmail.com (M. Nazim); aapkhan@gmail.com (A.A.P. Khan); rahmad5@jmi.ac.in (R. Ahmad).

Contents

Figure S1 DSC spectra of heating and cooling curves of (a) undoped and (b) Cr doped perovskite $CH_3NH_3PbBr_3$ nanocrystals…………………………………………………………………….S3

Figure S2 (a) EDX spectra with inset FESEM image and FESEM elemental images of (b) Br element, (c) C element, (d) Pb element, (e) N element of undoped perovskite nanocrystals…………………………………………………………………………...……….S4

Figure S3 TEM image of single nanorods (a) high resolution (b) low resolution with (c-e) FFT pattern and calculations……………………………………………………………………………...S5

Figure S4 XRD spectra of (a) undoped and Cr doped perovskite nanocrystals (b) high resolution peak of (100) plane, (b) high resolution peak of (200) and (210) planes, and (d) high resolution peak of (200) plane of perovskite phase…………………………………………………………………………S6

Figure S5 XRD spectra of Cr doped perovskite nanocrystals for phase stability at different temperatures……………………………………………………………………………………….S7

Figure S6 Tauc plots of (a) undoped and (b) Cr doped perovskite $CH_3NH_3PbBr_3$ nanocrystals……………………………………………………………………………………….S8

Figure S7 XPS analysis of (a) C 1s, (b) N 1s, and (c) histogram profile of various elements of Cr doped perovskite microrods……………………………………………………………….…………..S9

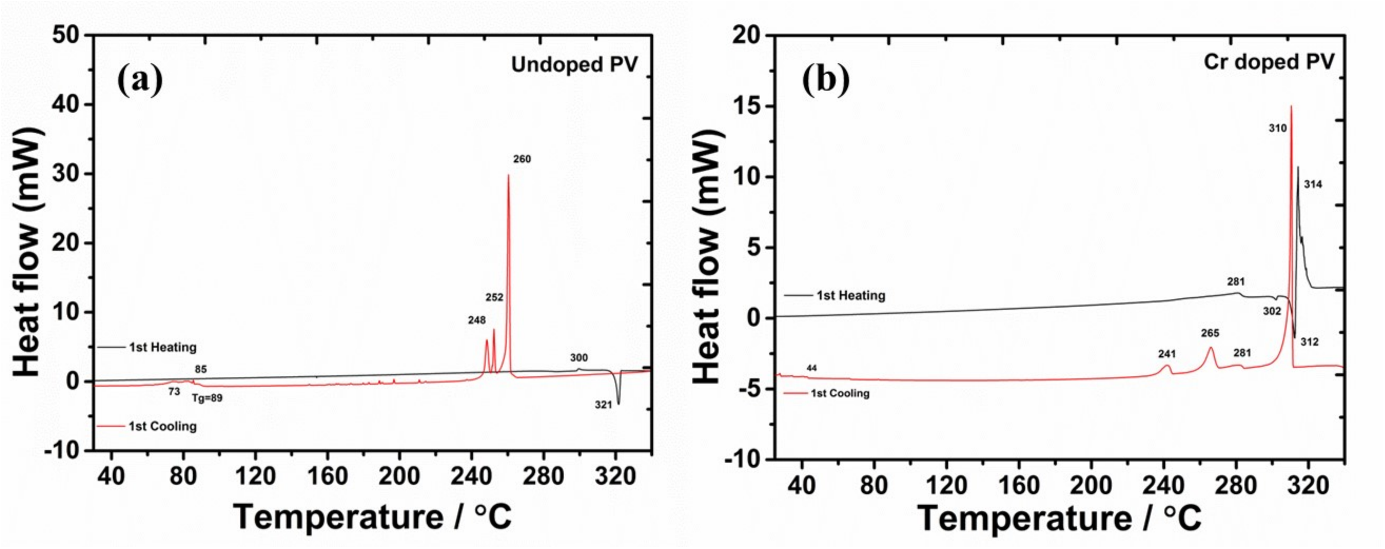

Figure S1 DSC spectra of heating and cooling curves of (a) undoped and (b) Cr doped perovskite $CH_3NH_3PbBr_3$ nanocrystals.

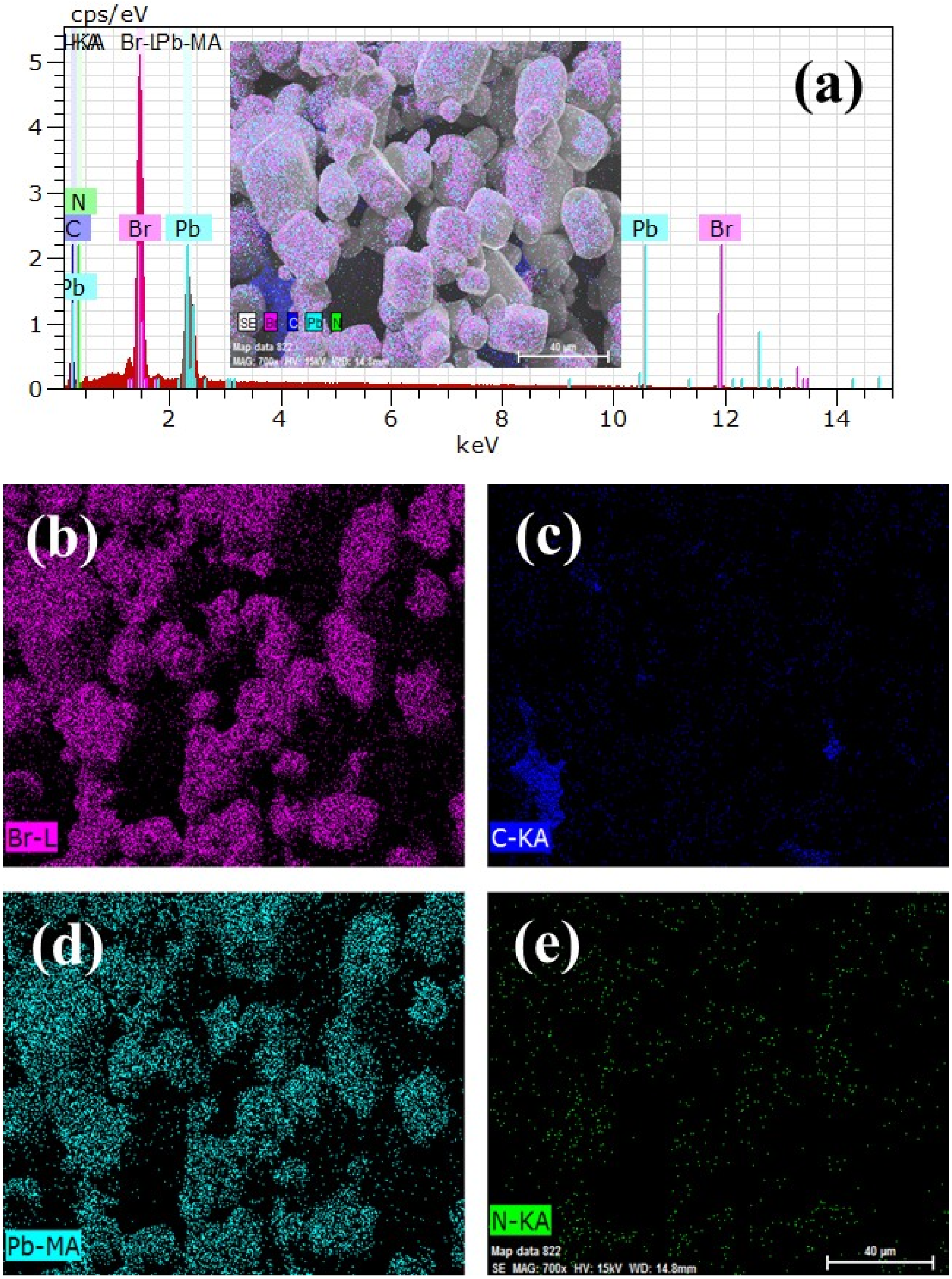

Figure S2 (a) EDX spectra with inset FESEM image and FESEM elemental images of (b) Br element, (c) C element, (d) Pb element, (e) N element of undoped perovskite nanocrystals.

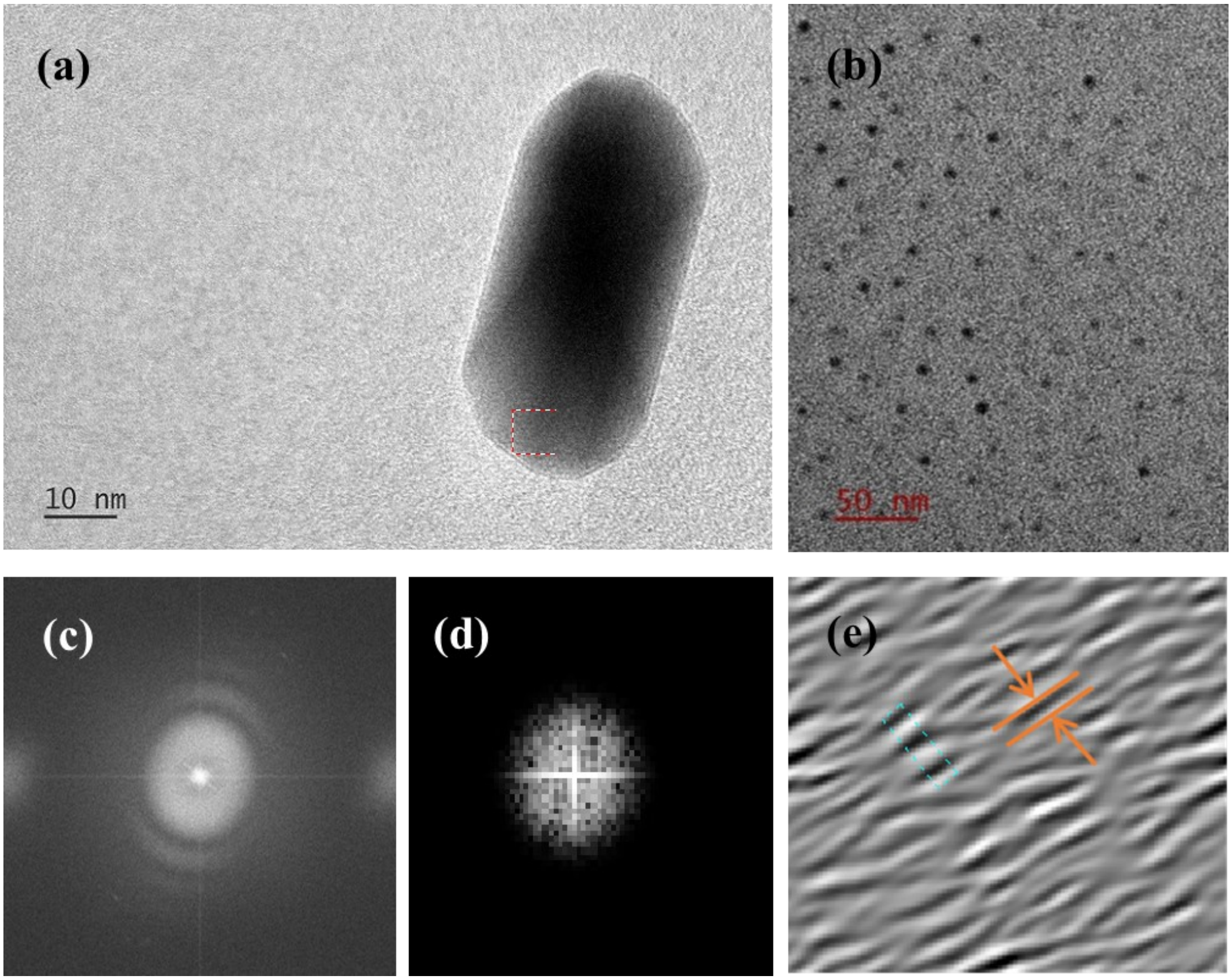

Figure S3 TEM image of single nanorods (a) high resolution (b) low resolution with (c-e) FFT pattern and calculations.

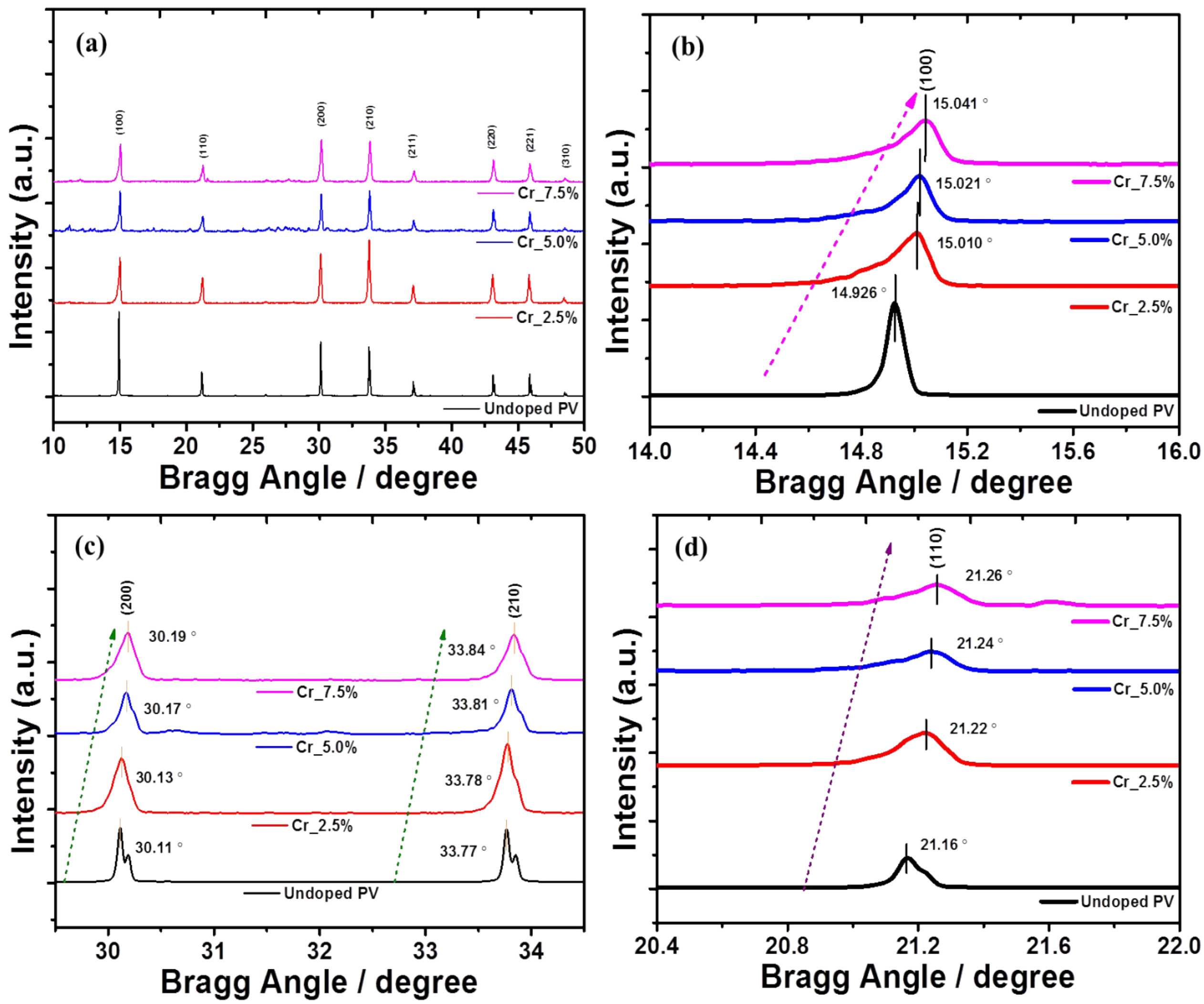

Figure S4 XRD spectra of (a) undoped and Cr doped perovskite nanocrystals (b) high resolution peak of (100) plane, (b) high resolution peak of (200) and (210) planes, and (d) high resolution peak of (110) plane of perovskite phase.

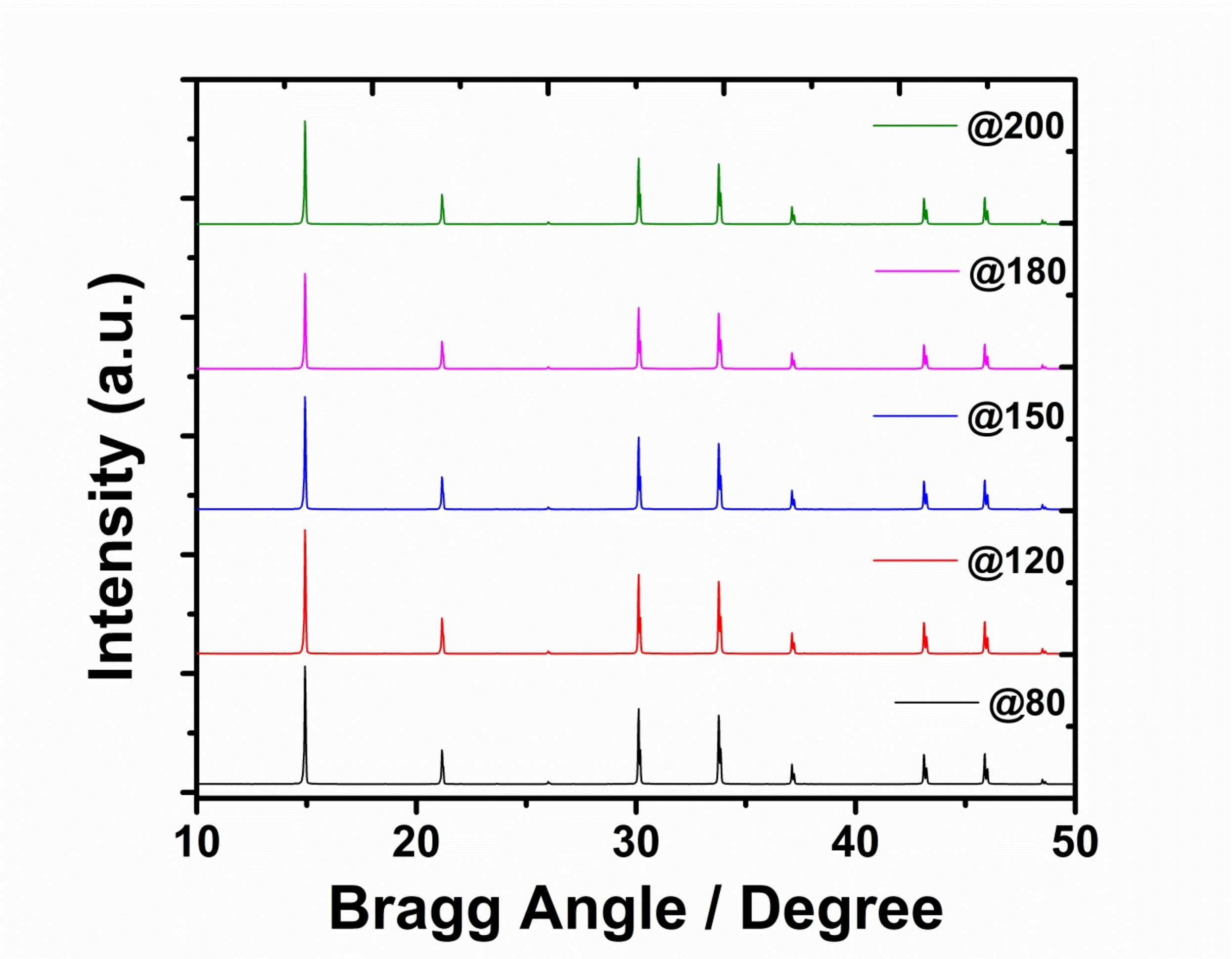

Figure S5 XRD spectra of Cr doped perovskite nanocrystals for phase stability at different temperatures.

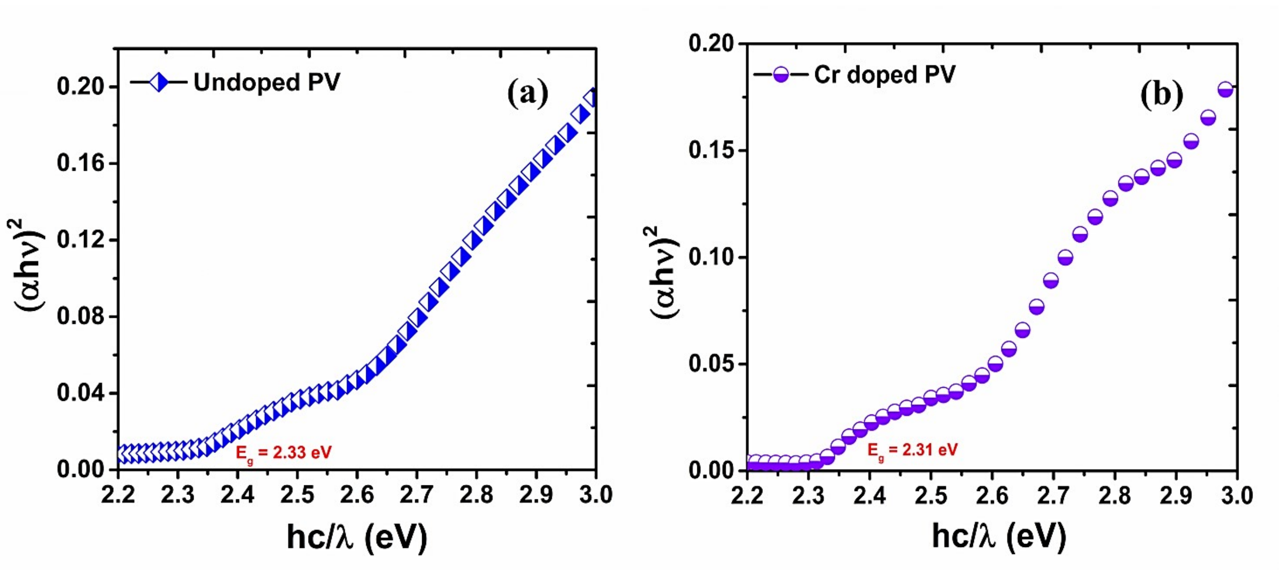

Figure S6 Tauc plots of (a) undoped and (b) Cr doped perovskite $CH_3NH_3PbBr_3$ nanocrystals.

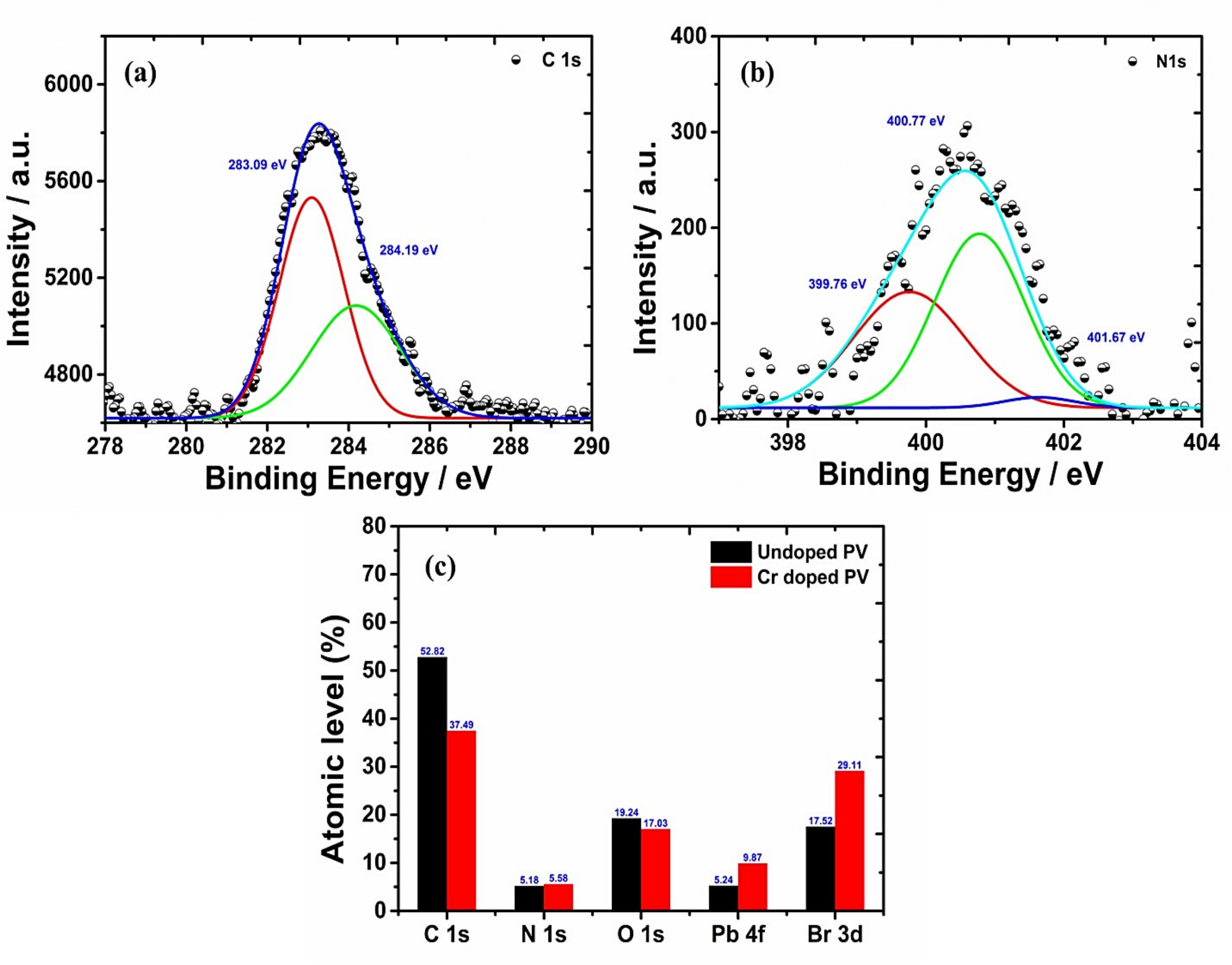

Figure S7 XPS analysis of (a) C 1s, (b) O 1s, and (c) histogram profile of various elements of Cr doped perovskite microrods.
